# An Intervention Delivered by Mobile Phone Instant Messaging to Increase Acceptability and Use of Effective Contraception Among Young Women in Bolivia: Randomized Controlled Trial

**DOI:** 10.2196/14073

**Published:** 2020-06-22

**Authors:** Ona L McCarthy, Carolina Aliaga, Maria Eugenia Torrico Palacios, Jhonny López Gallardo, Silvia Huaynoca, Baptiste Leurent, Phil Edwards, Melissa Palmer, Irrfan Ahamed, Caroline Free

**Affiliations:** 1 The London School of Hygiene and Tropical Medicine London United Kingdom; 2 CIES Salud Sexual Salud Reproductiva La Paz Bolivia; 3 International Planned Parenthood Federation/Western Hemisphere Region New York, NY United States

**Keywords:** Bolivia, contraception, mobile phone, cellphone, reproductive health, young adults

## Abstract

**Background:**

Although the most effective methods of contraception are available in Bolivia, unmet need for contraception among women aged 15 to 19 years is estimated to be 38% (2008), and the adolescent fertility rate is 71 per 1000 women (2016). Mobile phones are a popular mode to deliver health behavior support. We developed a contraceptive behavioral intervention for young Bolivian women delivered by mobile phone and guided by behavioral science. The intervention consists of short instant messages sent through an app over 4 months.

**Objective:**

This trial aimed to evaluate the effect of the intervention on young Bolivian women’s use of and attitudes toward the effective contraceptive methods available in Bolivia.

**Methods:**

This was a parallel group, individually randomized superiority trial with a 1:1 allocation ratio. Women were eligible if they were aged 16 to 24 years, owned a personal Android mobile phone, lived in La Paz or El Alto, reported an unmet need for contraception, and could read Spanish. The target sample size was 1310 participants. Participants allocated to the intervention had access to an app with standard family planning information and intervention messages. Participants allocated to the control group had access to the same app and control messages. Coprimary outcomes were use of effective contraception and acceptability of at least one method of effective contraception at 4 months. Secondary outcomes were use of effective contraception during the study, acceptability of the individual methods, service uptake, unintended pregnancy, and abortion. Process outcomes included knowledge, perceived norms, personal agency, and intention. Outcomes were analyzed using logistic and linear regression. We also asked participants about physical violence.

**Results:**

A total of 640 participants were enrolled, and 67.0% (429) of them contributed follow-up data for the coprimary outcome, the use of effective contraception. There was no evidence that use differed between the groups (33% control vs 37% intervention; adjusted odds ratio [OR] 1.19, 95% CI 0.80 to 1.77; *P*=.40). There was a borderline significant effect regarding acceptability (63% control vs 72% intervention; adjusted OR 1.49, 95% CI 0.98 to 2.28; *P*=.06). There were no statistically significant differences in any of the secondary or process outcomes. The intervention dose received was low. In the control group, 2.8% (6/207) reported experiencing physical violence compared with 1.9% (4/202) in the intervention group (Fisher exact test *P*=.75).

**Conclusions:**

This trial was unable to provide definitive conclusions regarding the effect of the intervention on use and acceptability of effective contraception because of under recruitment. Although we cannot strongly recommend implementation, the results suggest that it would be safe and may increase the acceptability of effective contraception if the intervention messages were offered alongside the download of the app.

**Trial Registration:**

ClinicalTrials.gov NCT02905526; https://clinicaltrials.gov/ct2/show/NCT02905526

## Introduction

Unintended pregnancy is associated with numerous poorer health outcomes for both women and their children [1-5]. Satisfying the unmet need for contraception is essential in helping women avoid unintended pregnancies, which requires an understanding of the barriers to use in specific settings [6]. A woman who has an unmet need for contraception is of reproductive age (15-49 years); is legally married, cohabiting, in a consensual union, or unmarried and sexually active; is not using any method of contraception; and is fecund and does not want to have a child (or another child) in the next 2 years or at all [6]. Modern contraceptive methods include oral contraceptives, injectables, intrauterine devices (IUDs), implants, the patch, the ring, male and female sterilization, male and female condoms and other barrier methods, modern fertility awareness methods, and emergency contraception [7]. *Effective* contraceptive methods are methods with less than 10% typical use failure rate at 12 months, that is, all modern methods besides condoms, other barrier methods, and modern fertility awareness methods [8,9].

In Bolivia, the latest Demographic and Health Survey (2008) reported that 85% of sexually active women aged between 15 and 19 years wanted to avoid a pregnancy, yet only 49% of these women reported using any contraceptive method [10]. Male condoms and the injection were the most common modern methods reported by this group (19.6% and 6.2%, respectively), with 2% reporting that they use withdrawal and 13% using periodic abstinence. A more recent survey (2016) reported that the adolescent fertility rate was 71 per 1000 women [11]. The 2016 survey found that among unmarried, sexually active women aged 15 to 19 years, an estimated 34% were not using any method of contraception [11], which was down from 52% in the 2008 survey [10]. Although unmet need may have changed since, the most recent data from 2008 suggest that unmet need among women aged 15 to 19 years was estimated to be 38% [10,12]. The (nonpermanent) effective methods available in Bolivia are oral contraceptive pills, IUDs, injectables, implants, and the patch. These methods are available for a fee (between US $1.50 and US $3.00) at the Centro de Investigación, Educación y Servicios (CIES), our partner organization, which operates 18 clinics in 7 of the 9 departments in the country. The public health care system provides only condoms to young people; however, young people usually buy condoms from private pharmacies.

The option of delivering health interventions by a mobile phone has gained popularity, particularly over the last decade [13-24]. Using mobile phones to deliver support to young people regarding sexual and reproductive health at the time of their choosing may be particularly useful, given the sensitivity of the topic. Randomized controlled trials have provided evidence that interventions delivered by a mobile phone can improve contraceptive use [25-27] and knowledge [25,28-30]. Other trials, however, have not found a beneficial effect [31-35]. The mixed evidence could be because of variability in the quality of the intervention development (eg, the target group may not have been adequately consulted), the content of the intervention (eg, the intervention content was not grounded in theory or behavioral science), and trial methodology.

The London School of Hygiene & Tropical Medicine (LSHTM) and CIES developed a contraceptive behavioral intervention for young Bolivian women delivered by mobile phone [36]. We developed the intervention guided by an established approach grounded in behavioral science [37]. The intervention is informed by the Integrated Behavioral Model (IBM) [38] and consists of short instant messages sent through CIES’s *Tú decides* app over 4 months. In this report, we present the results of the evaluation of the intervention by randomized controlled trial. The aim of the trial was to establish if the intervention increases young Bolivian women’s use and acceptability of the effective contraceptive methods.

## Methods

### Study Design and Participants

The methods reported in this section were first published in the trial protocol [39] and the statistical analysis plan [40].This was a parallel group, individually randomized superiority trial with a 1:1 allocation ratio that evaluated the effect of the intervention delivered by CIES’s app. Women were eligible to take part if they were aged 16 to 24 years, owned a personal Android mobile phone, lived in La Paz or El Alto, reported an unmet need for contraception (ie, are sexually active, not using effective contraception, and want to avoid a pregnancy), and could read Spanish. Participants must have been willing to download the app and receive messages about contraception on their mobile phone. The trial was promoted through CIES’s service delivery points in La Paz and El Alto, the CIES website, flyers distributed through CIES’s youth network, and social media sites. Participants provided informed consent through the secure web-based trial database and randomization system.

### Ethics Approval and Consent to Participate

The trial was granted ethical approval by LSHTM Interventions Research Ethics Committee on May 16, 2016, and by La Comisión de Ética de la Investigación del Comité Nacional de Bioética on September 20, 2016.

### Intervention and Control

The intervention was developed with young Bolivian people in 2015 to 2016, guided by a systematic protocol for the development of behavior change interventions [36]. The development process involved the following steps: (1) needs assessment (activities included establishing a project planning group, a literature search, focus group discussions, and interviews with the target group and interviews with local service providers), (2) specifying behavioral change to result from the intervention, (3) designing the intervention components by selecting behavior change methods, and (4) producing and refining the intervention content. The needs assessment revealed that young people in El Alto and La Paz were eager to receive information about contraception on their phone, lacked comprehensive knowledge about contraception, and expressed a range of negative beliefs about effective methods. The intervention messages were tested with young people and refined after each consultation in an iterative process until the context was acceptable to them.

The intervention provided accurate information about contraception, targeted the beliefs identified in the development phase that influence contraceptive use (eg, specific misconceptions about the side effects and health risks of contraception), and aimed to support young women in believing that they can influence their reproductive health. The messages contained 10 behavior change methods [41], adapted for delivery by mobile phone: belief selection, facilitation, anticipated regret, guided practice, verbal persuasion, tailoring, cultural similarity, arguments, shifting perspective, and goal setting. Participants allocated to the intervention group received 0 to 3 messages per day (a total of 183 messages) for 120 days. Please see the protocol [39] and the intervention development publication [36] for a more detailed description of the intervention.

The Tú decides app itself contained standard family planning information and no behavior change methods. Participants allocated to the intervention arm had access to the app and the intervention instant messages. Participants allocated to the control arm had access to the Tú decides app and 7 control instant messages about the importance of their participation and reminding them to contact the project coordinator if they change their number (which intervention participants also received). All participants who received usual care were free to seek any other support, whether existing or new.

### Allocation and Intervention Delivery

After providing informed consent, participants completed the baseline questionnaire through the database and randomization system. The allocation sequence was generated by the remote computer-based randomization software. Randomization occurred immediately after baseline data were submitted. All participants downloaded the app immediately after they submitted their baseline data. The messages commenced within 24 hours after participants downloaded the app.

### Protecting Against Bias

Owing to the nature of the intervention, participants would have been aware of the allocation soon after they started receiving the messages. Local research staff collecting outcome data were masked to allocation unless the participant revealed it to them. Researchers who analyzed the data were masked to treatment allocation.

### Outcomes

#### Coprimary Outcomes

The coprimary outcomes at 4 months post randomization were (1) self-reported current use of effective contraception and (2) the proportion of participants reporting that at least one method of effective contraception was acceptable. The primary outcome measure was constructed based on guidelines for measuring IBM constructs [38,42,43] and tested for face validity with the target group. The acceptability of each method was measured by the following stems: “Using the [method] ...causes infertility, ...causes unwanted side effects, ...is easy, ...is a good way to prevent pregnancy” and “I would recommend the [method] to a friend.” The IUD and implant include an additional stem: “The [method] insertion would not be a problem for me.” The response options for each stem were “strongly disagree,” “disagree,” “not sure,” “agree,” “strongly agree,” and “I do not know what the [method] is.” A method was acceptable if participants reported “agree” or “strongly agree” for all scales except for “...causes infertility” and “...causes unwanted side effects” stems, for which “disagree” or “strongly disagree” indicated acceptability [39].

#### Secondary Outcomes

The following secondary outcomes were self-reported: use of effective contraception at any time during the study, acceptability of each effective contraception method, attendance at a sexual health service during the study, unintended pregnancy during the study (the proportion reporting that they became pregnant and they did not want to become pregnant), and abortion during the study.

#### Process Outcomes

The following process outcomes were self-reported: knowledge of effective contraception, perceived norms, and personal agency in relation to using and communicating with partners about contraception, intention to use effective contraception, and intervention dose received.

### Data Collection

Data were collected at baseline and at 4 months post randomization using questionnaires. At baseline, we collected personal and demographic data and the coprimary outcome acceptability. At follow-up, we collected all outcomes and the following: if participants report using an effective method, where they obtained it; current pregnancy intention; whether they knew someone else who had also participated in the study and, if so, if they read each other’s messages; and to assess potential adverse outcomes, we asked participants if they have experienced physical violence since being in the study and if anything good or bad happened as a result of receiving the messages. An instant message that included a link to the database to complete the follow-up questionnaire was sent to all participants through the app 4 months after downloading the app. If participants did not complete the follow-up questionnaire themselves, local research staff unaware of participants’ allocation contacted them by telephone to collect their data.

### Sample Size

The trial was powered to detect a 10% absolute difference in the use of effective contraception between the intervention and control groups at 4 months. The 10% difference was based on a trial evaluating a postabortion mobile phone intervention, which found that 18% more women in the intervention arm than those in the control arm were using effective contraception at 4 months (64% vs 46%) [27]. We assumed that our trial would observe a smaller increase in contraceptive uptake, as it does not specifically involve women who had just had an abortion, who had already accessed services, and who may also have a greater intention to use contraception compared with the women in our trial. Therefore, we powered our trial to detect a smaller absolute difference of 10% uptake in effective contraception at 4 months.

The best estimate at the time of designing the trial was that the proportion of women aged 16 to 24 years in a partnership living in La Paz or El Alto using effective contraception was around 44% [44]. In total, 1048 participants provided 90% power to detect a 10% absolute difference, at the 5% significance level, assuming 44% use in the control group versus 54% in the intervention group (corresponding to an odds ratio [OR] of 1.49). Allowing for 20% loss to follow-up, the sample size was 1310.

### Statistical Analysis

The trial protocol was accepted for publication on November 3, 2017 [39], and the detailed statistical analysis plan was publicly released on November 7, 2017 [40]. Analyses were according to the randomized arm, and only participants with complete outcome data were included in the principal analysis. All statistical tests were two sided and were considered significant at the 5% level. The analysis was conducted using Stata 15 (StataCorp). Unmasking occurred on February 6, 2018, after the analyses outlined within the analysis plan were complete on masked data.

### Loss to Follow-Up and Missing Data

We used a chi-square test to investigate the evidence for whether losses to follow-up differed by trial arm. We used logistic regression to compare the baseline characteristics of participants who completed follow-up with those who did not. We investigated whether predictors of loss to follow-up differed by trial arm by testing for an interaction.

### Principal Analysis

#### Analysis of the Coprimary Outcomes

We compared the proportion that reported using a method of effective contraception or finding at least one method acceptable between the groups using logistic regression. We report the crude and adjusted OR along with the 95% CI and *P* value. The primary analysis was adjusted for the following prespecified baseline covariates: age (16-19 and 20-24 years), number of children (0 and ≥1), education level (university and other), and acceptability of effective contraception at baseline (at least one method acceptable and no methods acceptable) [39,40].

#### Analysis of the Secondary Outcomes

The analysis of the secondary outcomes was similar to the analysis of the primary outcome, although for the acceptability of the individual methods, the acceptability of that method at baseline replaced the acceptability of at least one method at baseline as a covariate.

#### Analysis of the Process Outcomes

The process outcomes comprised ordinal scales. Each scale was analyzed individually using ordered logistic regression to estimate proportional ORs. For knowledge, each correct answer received one point. The points were summed, and an overall score was produced for analysis. We used linear regression to test for a difference in the mean scores between the trial arms. To assess the *dose* of the intervention that the intervention participants received, we analyzed the number of messages that participants reported to have read (all, most, some, and none) and whether they stopped the messages.

### Additional Analyses

#### Sensitivity Analyses

We conducted 2 sensitivity analyses allowing for the missing data. In the first analysis (an *extreme case* analysis), we considered that all participants lost to follow-up did not use an effective method of contraception or did not find at least one method acceptable. In the second analysis, we adjusted for the main baseline predictors of missingness. Both sensitivity analyses were adjusted for the baseline covariates, as mentioned above.

#### Subgroup Analysis

We conducted an exploratory subgroup analysis for each coprimary outcome to determine if the intervention effect varied by baseline characteristics. The prespecified subgroups were age (split at the median), marital status (married/not married), number of children (0/≥1), geographical location (El Alto/La Paz), occupation (in education/other), and education level (university/other). Within the subgroups, we assessed the heterogeneity of treatment effect with a test for interaction [45-49]. We estimated ORs with 95% CIs for each subgroup.

#### Contamination

To assess the potential for contamination, we report the proportion of control group participants who reported that they read another participant’s messages and the proportion of intervention participants who reported that their messages were read by another participant.

#### Report of Physical Violence

We report the proportion of participants in each group who reported experiencing physical violence during the study.

## Results

### Recruitment, Randomization, and Exclusions

Between March 1, 2017, and July 29, 2017, there were 645 randomizations by the system. Follow-up ended on February 8, 2018. During the trial follow-up, we discovered that 3 participants enrolled and were randomized twice. For the 1 participant who was allocated to the same arm on both randomizations, we kept this participant in the analysis using the baseline data from their first record. For the 2 participants who were allocated to different arms, we excluded them from the analysis. This resulted in 640 participants included in the trial.

**Figure 1:**
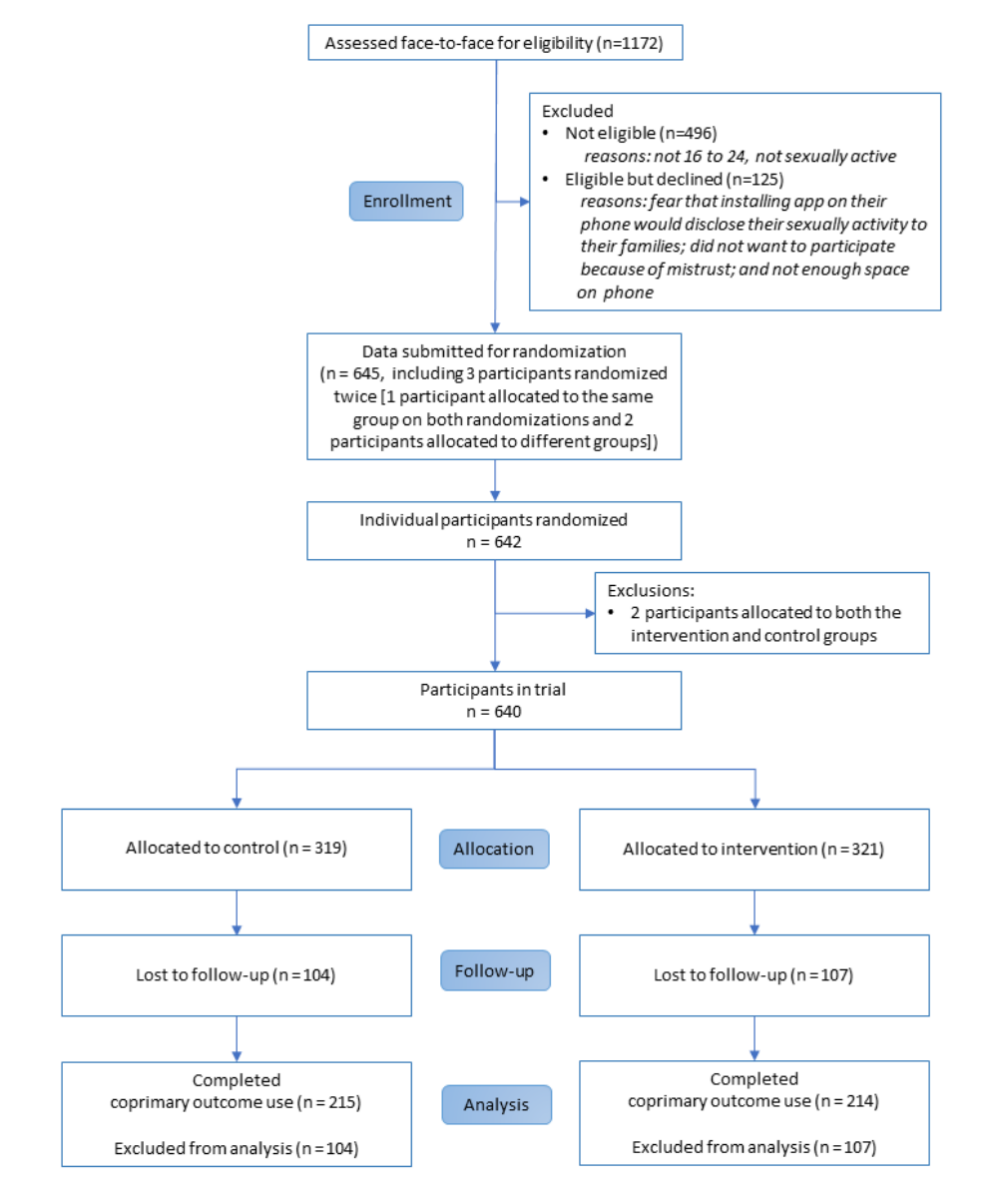
Trial flow diagram.

Of the 640 participants, 321 were allocated to the intervention arm and 319 participants were allocated to the control arm (Figure 1).

### Baseline Characteristics

The baseline characteristics of trial participants are reported in Table 1. Mean age was 20 years. In addition, 90.4% (579/640) of the participants did not have children, and only 8% (26/640) of the participants found at least one method of effective contraception acceptable. Acceptability was very low for the individual methods (1%-3%). Characteristics were similar between the 2 groups.

**Table 1:** Baseline characteristics.

Characteristic			Control (n=319)	Intervention (n=321)	All participants (N=640)
**Age (years)**					
	Mean (SD)		20.42 (2.6)	20.27 (2.9)	20.35 (2.6)
	**Range, n (%)**				
		16-19	150 (47.0)	165 (51.4)	315 (49.2)
		20-24	169 (52.9)	156 (48.6)	325 (50.8)
**Marital status, n (%)**					
	Married		14 (4.4)	18 (5.6)	32 (5.0)
	Not married		305 (95.6)	303 (94.4)	608 (95.0)
**Number of children, n (%)**					
	0		293 (91.9)	286 (89.1)	579 (90.5)
	1		16 (5.0)	21 (6.5)	37 (5.8)
	≥2		10 (3.1)	14 (4.4)	24 (3.8)
**Indigenous origin (ethnicity), n (%)**					
	Aymara		181 (56.7)	179 (55.8)	360 (56.3)
	Guarani		1 (0.3)	3 (0.9)	4 (0.6)
	Quechua		13 (4.1)	6 (1.9)	19 (3.0)
	Other indigenous		10 (3.1)	10 (3.1)	20 (3.1)
	No indigenous origin		114 (35.7)	123 (38.3)	237 (37.0)
**Occupation, n (%)**					
	Studying at school		61 (19.1)	58 (18.0)	119 (18.6)
	Studying at university		176 (55.2)	181 (56.4)	357 (55.8)
	Working		30 (9.4)	36 (11.2)	66 (10.3)
	Training		19 (6.0)	17 (5.3)	36 (5.6)
	Not working		4 (1.3)	4 (1.3)	8 (1.3)
	Working and studying		29 (9.1)	25 (7.8)	54 (8.4)
**Highest level of education completed, n (%)**					
	Primary		19 (6.0)	13 (4.1)	32 (5.0)
	Secondary		227 (71.2)	235 (73.2)	462 (72.2)
	University		68 (21.3)	62 (19.3)	130 (20.3)
	Technical		5 (1.6)	11 (3.4)	16 (2.5)
**Baseline method, n (%)**					
	None		240 (75.2)	257 (80.1)	497 (77.7)
	Male condom		46 (14.4)	36 (11.2)	82 (12.8)
	Female condom		9 (2.8)	4 (1.3)	13 (2.0)
	Other		24 (7.5)	24 (7.5)	48 (7.5)
**At least one effective method is acceptable, n (%)**					
	Yes		26 (8.2)	26 (8.1)	52 (8.1)
	No		293 (91.9)	295 (91.9)	588 (91.9)
**Pill acceptability, n (%)**					
	Yes		2 (0.6)	5 (1.6)	7 (1.1)
	No		317 (99.3)	316 (98.4)	633 (98.9)
**Intrauterine device** **acceptability, n (%)**					
	Yes		6 (1.9)	4 (1.3)	10 (1.6)
	No		313 (98.1)	317 (98.8)	630 (98.4)
**Injection acceptability, n (%)**					
	Yes		9 (2.8)	6 (1.9)	15 (2.3)
	No		310 (97.2)	315 (98.1)	625 (97.7)
**Implant acceptability, n (%)**					
	Yes		5 (1.6)	10 (3.1)	15 (2.3)
	No		314 (98.4)	311 (96.9)	625 (97.7)
**Patch acceptability, n (%)**					
	Yes		11 (3.5)	8 (2.5)	19 (3.0)
	No		308 (96.6)	313 (97.5)	621 (97.0)

### Loss to Follow-Up

Overall, 67.0% (429/640) participants completed the trial follow-up for the coprimary outcome use (control, n=215 and intervention, n=214; Figure 1), and 63.4% (406/640) participants completed the follow-up for the coprimary outcome acceptability (control, n=203 and intervention, n=203). Retention did not differ between the arms (215/319, 67.4% in the control arm and 214/321, 66.7% in the intervention arm; *P*=.84). Among the participants who completed the use coprimary outcome, the strongest predictor of retention was being aged 20 to 24 years (OR 1.33, 95% CI 0.96 to 1.86; *P*=.09). There was some evidence that the effect of this predictor differed by arm (interaction test *P*=.09). Detailed characteristics of participants who completed follow-up and those who did not are reported in Multimedia Appendix 1.

### Primary Outcomes

In the intervention arm, 37.4% (80/214) of participants reported the use of effective contraception compared with 33.5% (72/215) of participants in the control arm (Table 2). There was no evidence of a difference in use between the groups (crude OR 1.19, 95% CI 0.80 to 1.76; *P*=.40 and adjusted OR 1.19, 95% CI 0.80 to 1.77; *P*=.40).

### Secondary Outcomes

There were no significant differences in any of the secondary outcomes between the groups (Table 3).

In the intervention arm, 71.9% (146/203) of the participants reported that at least one method of contraception was acceptable compared with 62.6% (127/203) of participants in the control arm (Table 2). There was borderline evidence of a difference in acceptability between the groups (crude OR 1.53, 95% CI 1.01 to 2.33; *P*=.05 and adjusted OR 1.49, 95% CI 0.98 to 2.28; *P*=.06).

#### Process Outcomes

There were no significant differences in any of the process outcomes between the groups (Table 4).

**Table 2:** Coprimary outcomes.

Outcomes	Control			Intervention			Adjusted odds ratio (95% CI)		*P* value
	N	n (%)	N		n (%)				
Use of effective contraception^a^	215	72 (33.5)	214		80 (37.4)	1.19 (0.80 to 1.77)		.40	
At least one effective method is acceptable^a^	203	127 (62.6)	203		146 (71.9)	1.49 (0.98 to 2.28)		.06	

^a^Adjusted for age, number of children, education level, and acceptability at baseline.

**Table 3:** Secondary outcomes.

Outcomes	Control		Intervention		Adjusted odds ratio (95% CI)	*P* value
	N	n (%)	N	n (%)		
Pill acceptability^a^	206	52 (25.2)	207	59 (28.5)	1.19 (0.76 to 1.85)	.45
Intrauterine device acceptability^a^	206	43 (20.8)	206	55 (26.7)	1.37 (0.86 to 2.19)	.18
Injection acceptability^a^	208	79 (38.0)	207	93 (44.9)	1.30 (0.88 to 1.94)	.19
Implant acceptability^a^	206	63 (30.6)	205	65 (31.7)	1.03 (0.68 to 1.58)	.89
Patch acceptability^a^	208	95 (45.7)	208	109 (52.4)	1.31 (0.89 to 1.93)	.17
Long-acting reversible contraception acceptability^a^	204	106 (52.0)	205	120 (58.5)	1.31 (0.88 to 1.93)	.18
Effective contraceptive use during the 4 months^b^	210	76 (36.2)	206	73 (35.4)	0.94 (0.62 to 1.40)	.76
Service uptake^b^ (attended a service one or more times)	210	110 (52.4)	205	93 (45.4)	0.74 (0.50 to 1.10)	.14
Unintended pregnancy	319	1 (0.3)	321	0 (0.0)	N/A^c^	N/A
Induced abortion^d^	209	3 (1.4)	205	1 (0.5)	0.34 (0.01 to 4.24)^d^	.64

^a^Adjusted for age, number of children, education level, and the corresponding method’s acceptability at baseline.

^b^Adjusted for age, number of children, education level, and acceptability at baseline.

^c^N/A: not applicable.

^d^Unadjusted exact logistic regression.

**Table 4:** Process outcomes.

Process outcome		Control		Intervention		Proportional odds ratio^a^ (95% CI)		*P* value	
Knowledge of effective contraception, mean (SD)		4.3 (1.9)		4.5 (1.8)		0.17^b^ (−0.19 to 0.53)		.36	
**My friends would use the pill, IUD** ^c^ **, injection, or implant if they wanted to prevent pregnancy (N=205 for Control; N=202 for Intervention)** **, n (%)**						1.17 (0.73 to 1.88)		.51	
	Strongly disagree		2 (1.0)		0 (0)				
	Disagree		7 (3.4)		2 (1.0)				
	Not sure		29 (14.2)		13 (15.6)				
	Agree		159 (77.6)		161 (79.7)				
	Strongly agree		8 (3.9)		8 (4.0)				
**My friends would talk to their partner about contraception if they wanted to prevent a pregnancy (N=205 for Control; N=202 for Intervention)** **, n (%)**						1.33 (0.91 to 1.94)		.15	
	Strongly disagree		1 (0.5)		0 (0)				
	Disagree		17 (8.3)		14 (6.9)				
	Not sure		79 (38.5)		67 (33.2)				
	Agree		105 (51.2)		118 (58.4)				
	Strongly agree		3 (1.5)		3 (1.5)				
**If you wanted to use the pill, IUD, injection, or implant, how easy would it be for you to use it? (N=205** **for Control; N=202 for Intervention** **), n (%)**						0.98 (0.64 to 1.51)		.93	
	Very difficult		4 (2.0)		2 (1.0)				
	Difficult		24 (11.7)		25 (12.4)				
	Not sure		17 (8.3)		17 (8.4)				
	Easy		149 (72.7)		149 (73.8)				
	Very easy		11 (5.4)		9 (4.5)				
**If you wanted to talk to your partner about contraception, how easy would it be for you to talk to him? (N=205 for Control; N=202 for Intervention)** **, n (%)**						0.71 (0.48 to 1.06)		.09	
	Very difficult		1 (0.5)		7 (3.5)				
	Difficult		22 (10.7)		27 (13.4)				
	Not sure		33 (16.1)		25 (12.4)				
	Easy		128 (62.4)		136 (67.3)				
	Very easy		21 (10.2)		7 (3.5)				
**If you wanted to use the pill, IUD, injection, or implant, how certain are you that you could use it?** **(N=205 for Control; N=202 for Intervention),** **n (%)**						1.01 (0.66 to 1.55)		.97	
	Very certain I could not		2 (1.0)		2 (1.0)				
	Certain I could not		2 (1.0)		2 (1.0)				
	Not sure		34 (16.6)		36 (17.8)				
	Certain I could		151 (73.7)		143 (70.8)				
	Very certain I could		16 (7.8)		19 (9.4)				
**If you wanted to talk to your partner about contraception, how certain are you that you could talk to him? (N=204** **for Control; N=202 for Intervention** **), n (%)**						0.87 (0.58 to 1.30)		.49	
	Very certain I could not		0 (0)		4 (2.0)				
	Certain I could not		9 (4.4)		6 (3.0)				
	Not sure		46 (22.6)		45 (22.3)				
	Certain I could		131 (64.2)		137 (67.8)				
	Very certain I could		18 (8.8)		10 (5.0)				
**I intend to use the pill, IUD, injection, implant or patch (N=204** **for Control; N=202 for Intervention** **), n (%)**						0.74 (0.50 to 1.10)		.14	
	Strongly disagree		3 (1.5)		2 (1.0)				
	Disagree		14 (6.9)		16 (7.9)				
	Not sure		18 (8.8)		30 (14.9)				
	Agree		134 (65.7)		125 (61.9)				
	Strongly agree		35 (17.2)		29 (14.4)				
**Number of messages read (N=206), n (%)**						N/A^d^		N/A	
	All		N/A		13 (6.3)				
	Most		N/A		40 (19.4)				
	Some		N/A		94 (45.6)				
	None		N/A		59 (28.6)				
Proportion of intervention participants that stopped the intervention (N=205), n (%)		N/A		23 (11.2)		N/A		N/A	

^a^Estimated from ordered logistic regression.

^b^Mean difference.

^c^IUD: intrauterine device.

^d^N/A: not applicable.

#### Potential for Contamination

A total of 1.0% (2/209) of control participants said that they read the messages of someone else in the study. Moreover, 3.9% (8/205) of intervention participants said that someone else in the study read their messages.

#### Report of Physical Violence

A total of 2.9% (6/207) of participants in the control group and 2.0% (4/202) in the intervention group reported that they experienced physical violence since being in the study (Fisher exact test *P*=.75).

#### Intervention Dose

A total of 25.7% (53/206) of intervention participants reported that they read all or most of the intervention messages, with 28.6% (59/206) stating that they read none of the messages. In addition, 11.2% (23/205) of the participants reported that they stopped the intervention messages. Reasons intervention participants provided for not reading the messages or uninstalling the app were concerns about confidentiality, the app took up too much space on their phone, there were too many messages, and some messages were repetitive. In addition, 18.9% (39/206) of the intervention participants who answered the open-ended question “Did anything good or bad happened as a result of receiving the messages?” said that they did not receive any messages.

#### Participants’ Report of Anything Good or Bad That Happened During the Study

Almost half of the intervention participants that answered this question (97/206) reported something positive about the messages. The most common comment was that they learned new information. One participant said that they got pregnant and another said that they had *a scare due to carelessness*.

### Sensitivity Analyses

When we considered that participants who were lost to follow-up did not use an effective method or find an effective method acceptable, the effects observed in the principal analysis were reduced (use: OR 1.14, 95% CI 0.79-1.64; *P*=.48 and acceptability: OR 1.26, 95% CI 0.92-1.74; *P*=.15).

The strongest predictor of retention was being aged 20 to 24 years. Age was a baseline covariate, so the model in the second sensitivity analysis (adjusting for the main baseline predictors of missingness) is the same as the primary analysis model.

### Subgroup Analysis

**Figure 2:**
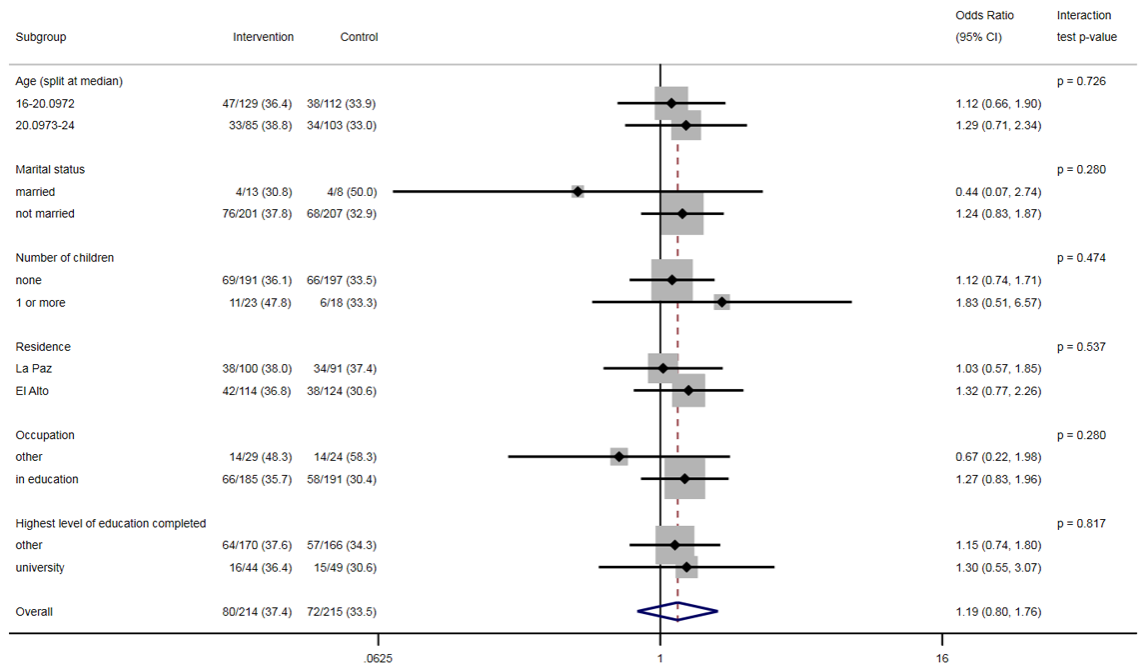
Intervention effect on the use of effective contraception by subgroups.

**Figure 3:**
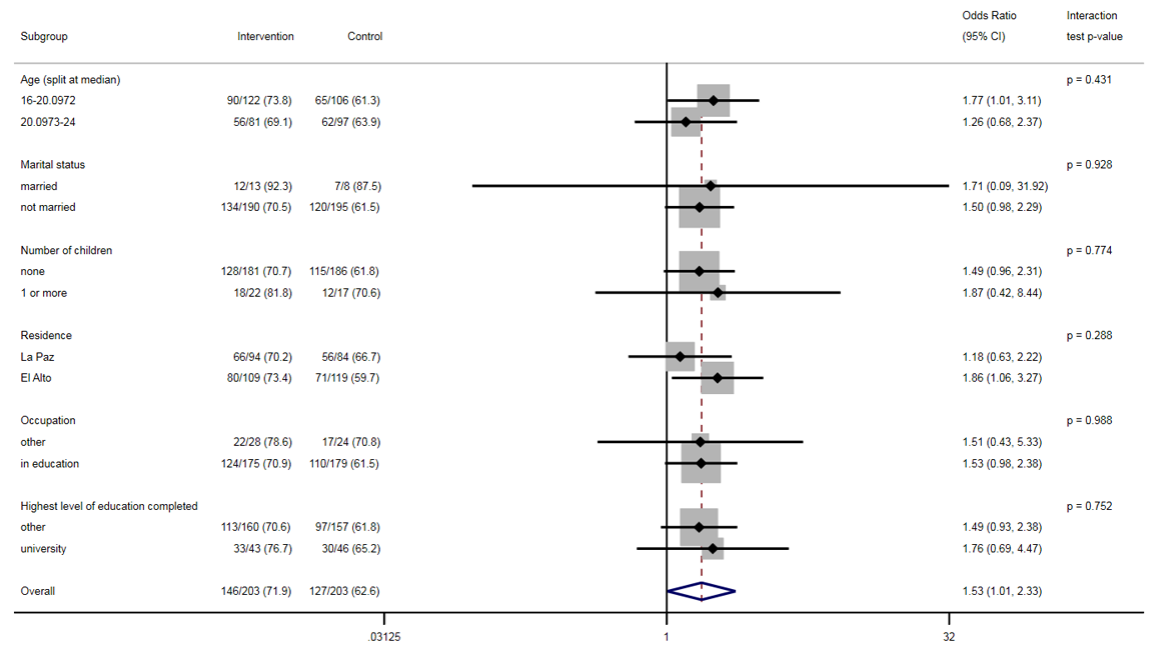
Intervention effect on the acceptability of effective contraception by subgroups.

There was no evidence that the effect of the intervention differed within the different levels of the subgroups (Figures 2 and 3).

## Discussion

### Principal Findings

The observed difference in contraceptive use between the groups (absolute difference=3.89%) was smaller than expected. Although the use was higher in the intervention group, the difference was not statistically significant. There was a borderline significant effect regarding the acceptability coprimary outcome, which favored the intervention group. No statistically significant differences between the groups in any of the secondary or process outcomes were observed. The intervention dose received was low, based on participants’ report.

### Strengths and Limitations

The main limitations of this trial were that we did not recruit to target and achieved less than 80% follow-up completion. Although effect estimates or both primary outcomes favored sending the intervention messages with the Tú decides app, the differences between the groups were smaller than what we expected. The recruitment and follow-up challenges meant that the trial was underpowered and therefore unable to produce unequivocal estimates regarding the effect of sending the intervention messages in addition to the app (with 429 participants, the trial had 54% power to detect a 10% absolute difference in use of effective contraception between the groups). Another limitation is in relation to the self-reported outcome measures. As they are self-reported, they are more likely to be biased than if the outcomes were objective, such as clinic-verified use of contraception.

Despite the limitations, this study had several strengths. Our trial database and randomization system generated and concealed the allocated and achieved well-balanced groups. There was no evidence that the intervention was associated with an increase in self-reported violence, a potential adverse outcome related to others viewing the messages, unwanted by the participant. We considered this a potential adverse outcome, given the stigma associated with sex before marriage in Bolivia [36]. However, we cannot determine the effect of the app on partner violence because both groups had access to it. Despite this, it is reassuring that the self-reported prevalence in this trial was low (2%).

### Comparisons With Existing Research

Trials evaluating contraceptive behavioral interventions delivered by mobile phone have had mixed results [25-35], with some showing an improvement in contraceptive use [25-27] and knowledge [25,28-30]. The results of this trial are not inconsistent with our trials of similar interventions among young people in Tajikistan [50] and Palestine [51]. In the Palestine trial, participants who received the intervention were more than twice as likely to find at least one method of contraception acceptable (OR 2.34, 95% CI 1.48-3.68; *P*<.001). There were also improvements in knowledge, acceptability of individual methods, perceived norms about friends using contraception, and intention to use contraception compared with the control group. In the Tajikistan trial, there was contamination between the intervention and control groups, and no differences were found between the groups. As with this Bolivian trial, the Tajik and Palestinian trials also did not suggest that the intervention was associated with an increase in violence.

### Implications of the Findings

The uncertainty regarding the efficacy of the intervention means that we cannot strongly recommend implementation in Bolivia. The low dose of the intervention is likely to have reduced the effect estimates. Only 25.7% (53/206) of the participants reported that they read all or most of the intervention messages, with 28.6% (59/206) reporting that they read none of the messages. This could have contributed to the smaller than anticipated observed differences. As we did not collect data on why these people did not read most of the messages, we do not know the reasons for this. A total of 47% (97/206) of intervention participants who answered the question “Did anything good or bad happen during the study?” (97/321, 30.2% of all intervention participants and 97/214, 45.3% of those who completed follow-up) reported something positive about the messages. Although participants in the development work were positive about the message content, sending the messages to the target group as they are intended to be sent (eg, over the entire 4 months) and then interviewing participants about their experience may have identified barriers to successful intervention receipt. We have done this in previous studies, but our timeline and resources did not allow for this in this trial.

Another possibility for the smaller than anticipated differences could be that the intervention is only moderately more effective than offering standard family planning information on a mobile app. It may be that offering good-quality family planning information on mobile app pages in this context is sufficient enough to improve the use of and attitudes toward contraception. However, the results suggest that the intervention messages could increase the acceptability of effective contraception if they were offered alongside the download of the Tú decides app and would not cause harm if done so.

It is difficult to determine exactly why we were unable to recruit to target. Some factors that likely contributed to the under-recruitment are (1) the trial promotion was not targeted to eligible people as much as it could have been, (2) young women were reticent to admit to being sexually active, (3) they did not want to have the app on their phone (although the development work indicated that this would not be a problem for them), and (4) they were not interested in taking part in a trial. Although the target sample size was much lower in the Tajik and Palestine trials (n=570 for each), this Bolivian trial actually recruited more participants and had a narrower inclusion criterion (women aged 16-24 years with an unmet need for contraception). Potential ways to improve recruitment in future trials of contraceptive behavioral interventions delivered by a mobile phone could be to promote the trial in settings where recruiters have a very good chance of accessing eligible people. In addition, only assessing potential participants on their own may improve the chance that they would admit to being sexually active.

In deciding how to analyze the scales, we thought it would be better to avoid false positives. For participants to score *acceptable* for a method, they must have chosen *agree* or *strongly agree* to the positively worded stems and *disagree* or *strongly disagree* to the negatively worded stems. To illustrate how stringent this definition of *acceptability* is, a participant could respond *strongly agree* to the statement that the method has unwanted side effects but could still use the method because the benefit of using it (avoiding a pregnancy) outweighs the risk [51]. This does not have implications on the effect of the intervention relative to the control. Nevertheless, the very low baseline acceptability of all methods highlights the need for interventions such as the one evaluated in this trial—interventions that provide accurate, nonjudgmental information about contraception and that address negative beliefs and misconceptions.

### Conclusions

This trial was unable to determine unequivocally if the intervention was effective at increasing the use and acceptability of effective contraception among young women with an unmet need in Bolivia. The intervention messages when delivered in addition to an app providing standard family planning information may moderately improve acceptability. Future research could first identify why around one-third of participants did not read the intervention messages and then evaluate the effect of the intervention on the use of contraception in different contexts.

## Appendix

Multimedia Appendix 1 Baseline characteristics by follow-up status.

## Abbreviations

CIES: Centro de Investigación, Educación y Servicios
IBM: Integrated Behavioral Model
IUD: intrauterine device
LSHTM: London School of Hygiene & Tropical Medicine
OR: odds ratio
