# Supplementary material for: An Intervention Delivered by Mobile Phone Instant Messaging to Increase Acceptability and Use of Effective Contraception Among Young Women in Bolivia: Randomized Controlled Trial
Source: J Med Internet Res. 2020 Jun 22;22(6):e14073. doi: 10.2196/14073 (PMC7338928; doi:10.2196/14073)
Supplement: Multimedia Appendix 1 [file jmir_v22i6e14073_app1.docx]

|  | | **Primary outcome use completers** | | **Primary outcome use non-completers** | | | | **All participants** | |
| --- | --- | --- | --- | --- | --- | --- | --- | --- | --- |
|  | | **Control**  N = 215  % (n) | **Intervention**  N = 214  % (n) | | **Control**  N = 104  % (n) | **Intervention**  N = 107  % (n) | **Control**  N = 319  % (n) | | **Intervention**  N = 321  % (n) |
| Age | mean [sd] | 20.69 [2.54] | 20.32 [2.61] | | 19.87 [2.50] | 20.17 [2.53] | 20.42 [2.56] | | 20.27 [2.58] |
|  | 16-19 | 42.33 (91) | 51.40 (110) | | 56.73 (59) | 51.40 (55) | 47.02 (150) | | 51.40 (165) |
|  | 20-24 | 57.67 (124) | 48.60 (104) | | 43.27 (45) | 48.60 (52) | 52.98 (169) | | 48.60 (156) |
| Marital status | married | 3.72 (8) | 6.07 (13) | | 5.77 (6) | 4.67 (5) | 4.39 (14) | | 5.61 (18) |
|  | not-married | 96.28 (207) | 93.93 (201) | | 94.23 (98) | 95.33 (102) | 95.61 (305) | | 94.39 (303) |
| Number of children | 0 | 91.63 (197) | 89.25 (191) | | 92.31 (96) | 88.79 (95) | 91.85 (293) | | 89.10 (286) |
|  | 1 | 5.58 (12) | 7.01 (15) | | 3.85 (4) | 5.61 (6) | 5.02 (16) | | 6.54 (21) |
|  | 2 or more | 2.79 (6) | 3.74 (8) | | 3.85 (4) | 5.61 (6) | 3.13 (10) | | 4.36 (14) |
| Indigenous origin (ethnicity) | Aymara | 59.53 (128) | 55.61 (119) | | 50.96 (53) | 56.07 (60) | 56.74 (181) | | 55.76 (179) |
|  | Guarani | 0.47 (1) | 0.47 (1) | | - | 1.87 (2) | 0.31 (1) | | 0.93 (3) |
|  | Quechua | 3.72 (8) | 2.34 (5) | | 4.81 (5) | 0.93 (1) | 4.08 (13) | | 1.87 (6) |
|  | other | 2.79 (6) | 2.80 (6) | | 3.85 (4) | 3.74 (4) | 3.13 (10) | | 3.12 (10) |
|  | none | 33.49 (72) | 38.79 (83) | | 40.38 (42) | 37.38 (40) | 35.74 (114) | | 38.32 (123) |
| Occupation | school | 15.35 (33) | 16.82 (36) | | 26.92 (28) | 20.56 (22) | 19.12 (61) | | 18.07 (58) |
|  | university | 57.21 (123) | 57.01 (122) | | 50.96 (53) | 55.14 (59) | 55.17 (176) | | 56.39 (181) |
|  | working | 9.77 (21) | 12.62 (27) | | 8.65 (9) | 8.41 (9) | 9.40 (30) | | 11.21 (36) |
|  | training | 5.12 (11) | 5.61 (12) | | 7.69 (8) | 4.67 (5) | 5.96 (19) | | 5.30 (17) |
|  | not working | 1.40 (3) | 0.93 (2) | | 0.96 (1) | 1.87 (2) | 1.25 (4) | | 1.25 (4) |
|  | university & working | 8.84 (19) | 6.07 (13) | | 4.81 (5) | 9.35 (10) | 7.52 (24) | | 7.17 (23) |
|  | school & working | 0.93 (2) | 0.47 (1) | | - | - | 0.63 (2) | | 0.31 (1) |
|  | training & working | 1.40 (3) | 0.47 (1) | | - | - | 0.94 (3) | | 0.31 (1) |
| Highest level of education completed | primary | 4.65 (10) | 3.74 (8) | | 8.65 (9) | 4.67 (5) | 5.96 (19) | | 4.05 (13) |
|  | secondary | 70.70 (152) | 73.36 (157) | | 72.12 (75) | 72.90 (78) | 71.16 (227) | | 73.21 (235) |
|  | university | 22.79 (49) | 20.56 (44) | | 18.27 (19) | 16.82 (18) | 21.32 (68) | | 19.31 (62) |
|  | technical | 1.86 (4) | 2.34 (5) | | 0.96 (1) | 5.61 (6) | 1.57 (5) | | 3.43 (11) |
| Baseline method | none | 73.95 (159) | 79.44 (170) | | 77.88 (81) | 81.31 (87) | 75.24 (240) | | 80.06 (257) |
|  | male condom | 14.88 (32) | 10.75 (23) | | 13.46 (14) | 12.15 (13) | 14.42 (46) | | 11.21 (36) |
|  | female condom | 3.26 (7) | 1.40 (3) | | 1.92 (2) | 0.93 (1) | 2.82 (9) | | 1.25 (4) |
|  | calendar | 1.40 (3) | 2.80 (6) | | - | 0.93 (1) | 0.94 (3) | | 2.18 (7) |
|  | withdrawal | 0.47 (1) | - | | 0.96 (1) | - | 0.63 (2) | | - |
|  | male & female condom | 0.93 (2) | - | | - | - | 0.63 (2) | | - |
|  | calendar & withdrawal | - | 0.47 (1) | | - | 0.93 (1) | - | | 0.62 (2) |
|  | other | - | - | | 5.77 (6) | 3.74 (4) | 0.63 (2) | | - |
| At least one effective method is acceptable | yes | 8.37 (18) | 9.35 (20) | | 7.69 (8) | 5.61 (6) | 8.15 (26) | | 8.10 (26) |
|  | no | 91.63 (197) | 90.65 (194) | | 92.31 (96) | 94.39 (101) | 91.85 (293) | | 91.90 (295) |
| Pill acceptability | yes | 0.93 (2) | 1.87 (4) | | - | 0.93 (1) | 0.63 (2) | | 1.56 (5) |
|  | no | 99.07 (213) | 98.13 (210) | | 100 (104) | 99.07 (106) | 99.37 (317) | | 98.44 (316) |
| IUD ^a^ acceptability | yes | 1.40 (3) | 1.40 (3) | | 2.88 (3) | 0.93 (1) | 1.88 (6) | | 1.25 (4) |
|  | no | 98.60 (212) | 98.60 (211) | | 97.12 (101) | 99.07 (106) | 98.12 (313) | | 98.75 (317) |
| Injection acceptability | yes | 2.70 (6) | 1.87 (4) | | 2.88 (3) | 1.87 (2) | 2.82 (9) | | 1.87 (6) |
|  | no | 97.21 (209) | 98.13 (210) | | 97.12 (101) | 98.13 (105) | 97.18 (310) | | 98.13 (315) |
| Implant acceptability | yes | 1.86 (4) | 3.27 (7) | | 0.96 (1) | 2.80 (3) | 1.57 (5) | | 3.12 (10) |
|  | no | 98.14 (211) | 96.73 (207) | | 99.04 (103) | 97.20 (104) | 98.43 (314) | | 96.88 (311) |
| Patch acceptability | yes | 3.72 (8) | 2.80 (6) | | 2.88 (3) | 1.87 (2) | 3.45 (11) | | 2.49 (8) |
|  | no | 96.28 (207) | 97.20 (208) | | 97.12 (101) | 98.13 (105) | 96.55 (308) | | 97.51 (313) |

^a^ IUD = Intrauterine device
